# Hyperelastic Material Properties of Mouse Skin under Compression

**DOI:** 10.1371/journal.pone.0067439

**Published:** 2013-06-18

**Authors:** Yuxiang Wang, Kara L. Marshall, Yoshichika Baba, Gregory J. Gerling, Ellen A. Lumpkin

**Affiliations:** 1 Department of Systems and Information Engineering, University of Virginia, Charlottesville, Virginia, United States of America; 2 Department of Dermatology, Columbia University College of Physicians and Surgeons, New York, New York, United States of America; 3 Department of Physiology and Cellular Biophysics, Columbia University College of Physicians and Surgeons, New York, New York, United States of America; Oak Ridge National Laboratory, United States of America

## Abstract

The skin is a dynamic organ whose complex material properties are capable of withstanding continuous mechanical stress while accommodating insults and organism growth. Moreover, synchronized hair cycles, comprising waves of hair growth, regression and rest, are accompanied by dramatic fluctuations in skin thickness in mice. Whether such structural changes alter skin mechanics is unknown. Mouse models are extensively used to study skin biology and pathophysiology, including aging, UV-induced skin damage and somatosensory signaling. As the skin serves a pivotal role in the transfer function from sensory stimuli to neuronal signaling, we sought to define the mechanical properties of mouse skin over a range of normal physiological states. Skin thickness, stiffness and modulus were quantitatively surveyed in adult, female mice (*Mus musculus*). These measures were analyzed under uniaxial compression, which is relevant for touch reception and compression injuries, rather than tension, which is typically used to analyze skin mechanics. Compression tests were performed with 105 full-thickness, freshly isolated specimens from the hairy skin of the hind limb. Physiological variables included body weight, hair-cycle stage, maturity level, skin site and individual animal differences. Skin thickness and stiffness were dominated by hair-cycle stage at young (6–10 weeks) and intermediate (13–19 weeks) adult ages but by body weight in mature mice (26–34 weeks). Interestingly, stiffness varied inversely with thickness so that hyperelastic modulus was consistent across hair-cycle stages and body weights. By contrast, the mechanics of hairy skin differs markedly with anatomical location. In particular, skin containing fascial structures such as nerves and blood vessels showed significantly greater modulus than adjacent sites. Collectively, this systematic survey indicates that, although its structure changes dramatically throughout adult life, mouse skin at a given location maintains a constant elastic modulus to compression throughout normal physiological stages.

## Introduction

As our primary interface with the environment, the skin plays an essential protective role in shielding the body from insults, including mechanical forces, chemicals and radiation. Skin is a stratified squamous epithelium comprising epidermal, dermal and hypodermal layers, which cover muscle, nerves and bone [1]. The skin's complex mechanical properties are essential for fulfilling its protective role. This tough yet flexible matrix is capable of withstanding continuous mechanical stress while accommodating changes including dynamic insults and organism growth.

The skin is a non-linear, hyperelastic material [2] that exhibits time-dependent viscoelastic relaxation and creep. These properties are set by elastin, proteoglycan, collagen and interstitial fluid [3], [4]. A better understanding of these intricate mechanical properties is needed to identify mechanisms of skin aging and sensory signaling, and to facilitate the development of new surgical procedures, transcutaneous drug delivery systems and personal care products.

Measuring the skin's many mechanical dimensions is a complex undertaking. Mechanical and structural properties, including thickness and elasticity, change with age and between body sites [5]–[9]. Furthermore, as a multi-layer structure rather than a homogeneous continuum, skin is expected to behave radically different under compression and tension. Skin mechanics have been measured using tension, compression, torque loading and indentation; however, tension tests are most extensively employed [6], [10]–[12]. For example, in uniaxial tensile tests with human cadaver skin, stress-strain curves were found to be linear under small deformations and non-linear at larger strain levels [6], [13]. By contrast, few studies have performed mechanical measurements of skin under compression. In one case, compression was applied to pig skin, though with only small deformations on a single specimen [2].

Compression is a clinically relevant regime in which to study skin mechanics. For instance, patient bedsores caused by long-term compression can lead to serious morbidity and mortality. This regime is also essential to studies of touch sensation, since objects encountered during daily tasks compress the skin's surface. Despite its relevance to naturalistic and pathological stimuli, no quantitative survey of compressive skin mechanics has been reported for any mammalian species.

To fill this gap, we analyzed the skin's hyperelastic properties under compression. As skin structure and physiology changes throughout an animal's life, we sought to compare skin biomechanical properties in a population of animals over a range of normal physiological states. We focused on mouse skin, since mouse models are extensively used to analyze mechanisms of skin biology and pathophysiology, including aging and UV-induced skin damage [14], [15]. Moreover, sensory mechanisms underlying touch and pain are widely analyzed by recording from mouse cutaneous nerves while applying thermal, chemical or mechanical stimuli to the skin [16]–[21]. As the skin serves a pivotal role in the transfer function from sensory stimuli to neuronal signaling, we sought to define the mechanical properties of mouse skin.

## Results and Discussion

**Figure 1:**
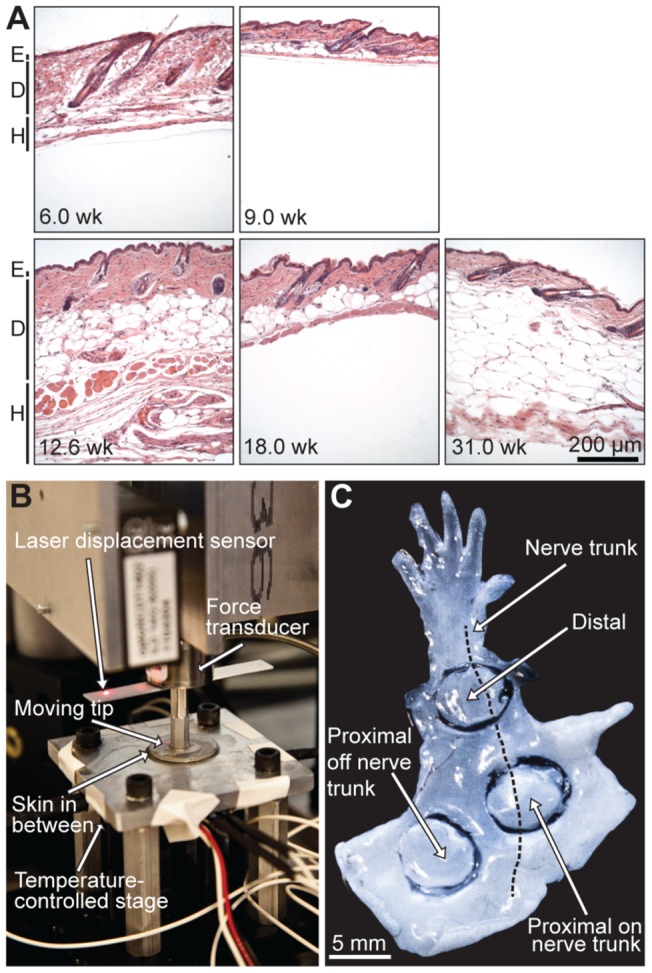
Skin histology and compression test apparatus. (**A**) Hemotoxylin and eosin (H&E) staining of skin sections from mice at 6.0, 9.0, 12.6, 18.0 and 31.0 weeks. Each specimen was harvested from the proximal hind limb adjacent to the saphenous nerve and vein. Skin layers are indicated at left (E: epidermis; D: dermis; H: hypodermis). (**B**) Uniaxial compression test apparatus. (**C**) Hind-limb skin from a 10-week-old mouse illustrates three sampling locations tested. Hair was removed with a depilatory cream prior to dissection. The lack of pigmentation indicates that this mouse was in the resting stage of the hair cycle.

To determine whether changes in skin mechanics accompany normal growth, we surveyed mice over an age range corresponding from adolescence (puberty onset) to middle age (mid-point of a typical mouse lifespan). We also compared three adjacent anatomical locations widely used for neurosensory studies [16]–[20]. Over the age range examined (5.7–34.3 weeks), mice exhibit two synchronous waves of hair growth followed by mosaic hair cycling [22]–[24]. Thus, mouse age and degree of skin pigmentation was used to classify resting (telogen) versus active (anagen/catagen) hair-cycle stages [25]. Histological examination showed that epidermal thickness was similar among groups (Figure 1A); however, dermal fat thickness varied dramatically among age groups and hair-cycle stages.

### A method for measuring skin material properties under compression

We performed uniaxial compression tests on freshly excised, full-thickness skin specimens. A custom-built test apparatus (Figure 1B) delivered controlled displacements, linearly ramped into the skin at a velocity of 10 μm·s^−1^, to collect force-displacement curves. These data were then translated into stress and strain curves.

For many materials, two key measures of elasticity–elastic stiffness and elastic modulus–can be derived from the linear slopes of force-displacement and stress-strain curves, respectively. Because skin is a hyperelastic material, these curves are instead non-linear. We therefore fit experimental data to modified exponential functions to approximate two hyperelastic parameters defined in detail in *Materials and Methods*. First, force-displacement curves were fit to estimate the stiffness coefficient (p). Intuitively, this paramater relates to the skin's resistance to deformation during displacement. For simplicity, we refer to the stiffness coefficient p as *skin stiffness*. Second, we fit stress-strain curves to derive the modulus coefficient (q). Modulus also relates to the skin's resistance to deformation but is scaled by the thickness of the specimen. For simplicity, we refer to the modulus coefficient q as *elastic modulus*.

Circular punch biopsies (6 mm in diameter) were tested from female mice differing in body weight, maturity and hair-cycle stage (n = 105 specimens from 24 mice). Three hind-limb sites were chosen because they differ in thickness and underlying fascial structures (Figure 1C). First, we compared distal hind-limb skin, which is thin, with thicker skin from two sites on the proximal hind limb. Second, to determine whether fascial structures impact skin mechanics, we compared skin sites directly over the saphenous nerve trunk and vein (proximal, on nerve trunk; NT) with those adjacent to the saphenous nerve and vein (proximal, off nerve trunk; OffNT).

**Figure 2:**
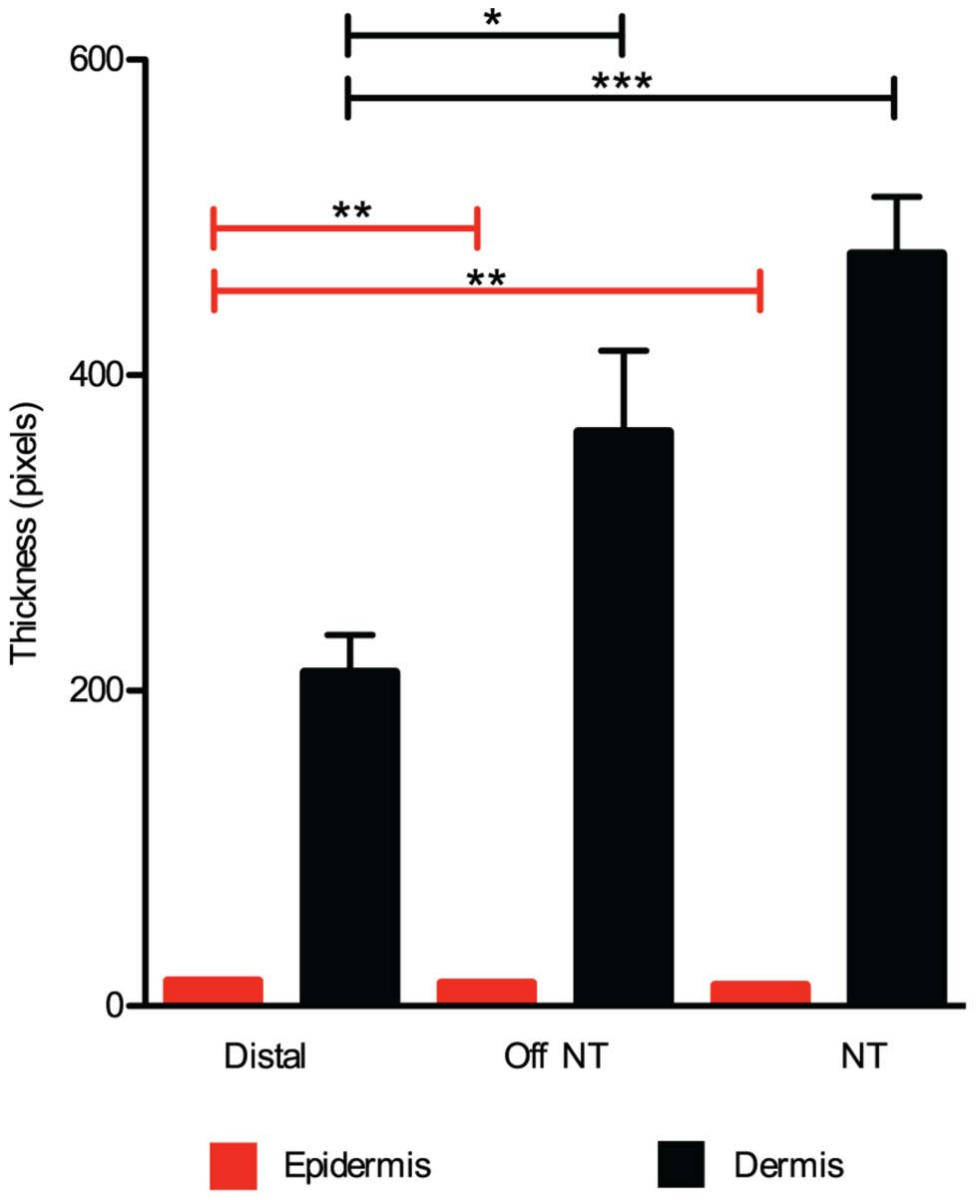
Histomorphometric analysis of epidermal and dermal thickness. Thickness values for epidermis (red) and dermis (black) measured from H&E-stained sections of mouse skin. Specimens were harvested from the hind limb in three areas: distal, proximal off nerve trunk (OffNT) and proximal on nerve trunk (NT). The distal site had a significantly thinner dermis than OffNT and NT (n = 9 sections from three mice per area; *P = 0.016 and ***P<0.0001, respectively; Student's two-tailed unpaired *t* test). By contrast, distal epidermis was slightly but significantly thicker than OffNT and NT epidermis (**P<0.002).

We performed quantitative histomorphometery on skin paraffin sections to determine whether the thickness of the epidermis or dermis differed between these three hind-limb sites. As fixation and staining procedures can alter the absolute values of tissue measurements, we compared the relative proportions of epidermal and dermal layers [26]. For all sites, we noted that the dermis was dramatically thicker than the epidermis, representing at least 93% of the combined epidermal and dermal thickness. We found that the dermis was on average 44–58% as thick in distal specimens compared with proximal NT and OffNT specimens (P<0.0001 and P = 0.016, respectively; Student's *t* test; Figure 2). By contrast, we observed a slight (12%) but significant increase in the thickness of nucleated epidermal layers in distal skin specimens compared with either proximal group (Figure 2). Thus, we conclude that dermal thickness primarily accounts for the differences observed between these skin sites.

To accurately measure the thickness (*l_0_*) of freshly excised skin specimens, we developed a procedure that compares probe position at skin contact (as measured by a contact force) to the stage position (see *Materials and Methods*). This provides highly repeatable measurements that overcome the limitations of caliper meaurements [11], which are more sensitive to observer error.

**Figure 3:**
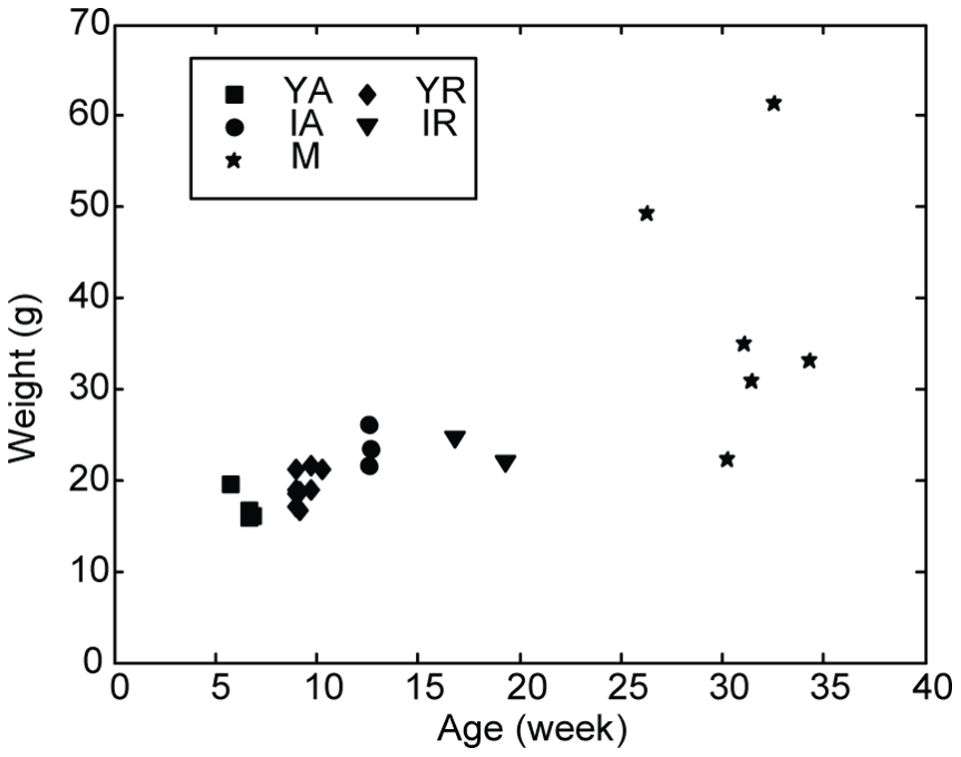
Plot of body weight versus age for female mice. Symbols denote animal grouping based on maturity level and hair-cycle stage [25]: **YA**: young adult, active cycling (5.7–6.9 weeks), **YR**: young adult, resting (9.0–10.3 weeks); **IA**: intermediate active (12.6–12.7 weeks); **IR**: intermediate resting (16.9–19.3 weeks); **M:** mature (26.3–34.3 weeks).

We next analyzed five independent variables [body weight (Figure 3), hair-cycle stage, maturity level, skin site and individual animal] to assess their impact on skin thickness, skin stiffness and elastic modulus. Over the entire population, these three biomechanical parameters were highly variant (thickness: 278±102 μm; CV = 0.368; stiffness coefficient p: 42.06±11.79 mm^−1^; CV = 0.280; elastic modulus coefficient q: 10.77±2.03; CV = 0.188). These data suggest that, like human skin, the mechanical properties of mouse skin change throughout adulthood.

### Body weight sets skin material properties in mature mice

**Figure 4:**
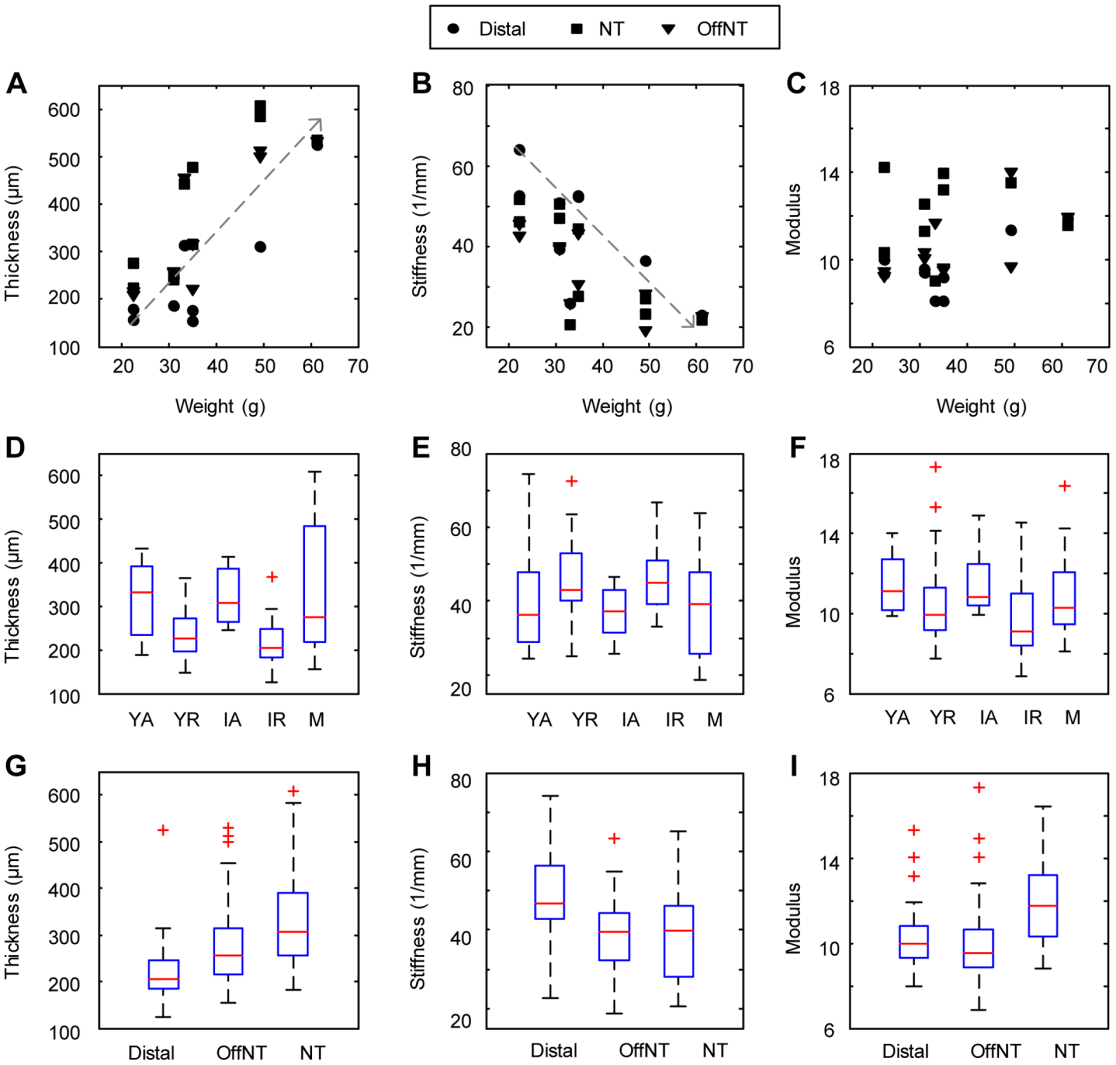
Skin properties under compression measured across weight, hair cycle and skin location. Body weight for mature mice plotted versus skin thickness (**A**), stiffness (**B**) and elastic modulus (**C**). Linear regression (dotted lines) indicate that body weight is significantly correlated with skin thickness (r = 0.793, p-value<10^−6^), inversely correlated with skin stiffness (r = −0.717, p-value<10^−3^) and uncorrelated with modulus. Skin mechanical parameters are shown with respect to hair cycle stages (**D–F**) and skin site (**G–I)**. Statistical significance was assessed by Pearson test of correlation and unpaired Student's *t* test and results are given in Tables 1 and 2. For all plots, boxes range from lower to upper quartiles, red lines within boxes indicate medians, black lines outside of boxes indicate max. and min. values and red (+) indicate outliers beyond 1.5 interquartile range.

Since cutaneous fat is added as an animal gains weight, we first asked whether body weight governs skin mechanics. For mature animals (26.3–34.3 weeks), body weight was positively correlated with skin thickness (Figure 4A). By contrast, body weight inversely correlated with skin stiffness for these mice (Figure 4B). The opposing changes in thickness and stiffness resulted in a consistent elastic modulus (Figure 4C), since modulus (q) is the product of thickness and stiffness [Eqn. (7)].

By contrast, no correlations between body weight and skin mechanical parameters were observed among animals in either young adult (5.7–10.3 weeks) or intermediate (12.6–19.3 weeks) age groups (Table 1). Although body weight differed by as much as 65% between young adult and intermediate mice, we noted that the variability was less (CV = 0.15) than in mature mice (CV = 0.37). This might contribute to a lack of correlation between weight and skin parameters in the first two groups. Based on these findings, we conclude that body weight governs skin thickness and stiffness in mature mice (>26 weeks of age) and we hypothesized that other physiological factors govern skin material properties in mice less than 20 weeks old.

**Table 1:** Relationship of body weight to skin properties (Pearson correlation).

		P-value			Correlation coefficient (r)		
		Mature	Active	Resting	Mature	Active	Resting
Thickness	**Distal**	*0.003*	0.124	0.230	*0.862*	0.589	−0.298
	**OffNT**	*0.001*	0.280	0.688	*0.870*	0.529	−0.102
	**NT**	*0.003*	0.719	0.651	*0.835*	−0.152	−0.114
	**All sites**	*0.000*	0.336	0.362	*0.793*	0.215	−0.127
Stiffness (p)	**Distal**	*0.026*	0.267	0.750	−*0.727*	−0.447	0.081
	**OffNT**	*0.004*	0.376	0.226	−*0.813*	−0.446	−0.301
	**NT**	*0.014*	0.657	0.253	−*0.744*	0.187	0.284
	**All sites**	*0.000*	0.220	0.781	−*0.717*	−0.272	0.039
Modulus (q)	**Distal**	*0.044*	0.911	0.354	0.679	−0.047	−0.232
	**OffNT**	0.065	0.754	0.155	0.603	−0.165	−0.350
	**NT**	0.478	0.981	0.252	0.255	0.010	0.285
	**All sites**	*0.039*	0.786	0.513	0.386	−0.062	−0.091

Correlations of mechanical properties (thickness, stiffness and modulus) with body weight are shown. Body site and age group of the comparison are indicate by row and column label, respectively. Bold font indicates group and italics denote p-values <0.05, or |r|>0.7.

### Skin mechanical properties cycle with hair growth rather than age

Since mice undergo two synchronous hair cycles between 5 and 15 weeks of age, we next investigated the effect of hair cycle on the skin's material properties. A W-shape trend in skin thickness was observed as mice matured (Figure 4d). For both young and intermediate ages, mean skin thickness was 33.7% higher during active hair-cycle phases (anagen/catagen) compared with resting (telogen) skin (Table 2; p≤0.007 for all comparisons; Student's unpaired *t* tests). These quantitative results corroborate previous histological reports of dramatic increases in skin thickness at anagen onset [25]. This striking expansion could be caused by increased dermal fat (Figure 1) or hair follicle lengths [25]. We found that skin stiffness (p) also varied over hair cycles (Figure 4E). We observed an M-shape trend that opposed thickness changes: skin was significantly less stiff in active phases than in resting phases. Since stiffness and thickness varied inversely over the hair cycle, the elastic modulus (q) was not significantly correlated with hair-cycle stage (Figure 4F and Table 2).

**Table 2:** Student's *t* tests comparing skin properties across groups and skin sites.

		Hair cycle or maturity level				Skin site		
		YR	IA	IR	M		OffNT	NT
Thickness	**YA**	*0.001*	0.747	*0.007*	0.581	**Distal**	*0.007*	*0.000*
	**YR**		*0.000*	0.249	*0.000*	**OffNT**		*0.022*
	**IA**			*0.002*	0.795			
	**IR**				*0.012*			
Stiffness (p)	**YA**	0.154	0.563	0.199	0.557	**Distal**	*0.000*	*0.000*
	**YR**		*0.031*	0.704	*0.006*	**OffNT**		0.790
	**IA**			*0.030*	0.928			
	**IR**				*0.033*			
Modulus (q)	**YA**	0.138	0.824	*0.030*	0.449	**Distal**	0.879	*0.000*
	**YR**		0.155	0.310	0.363	**OffNT**		*0.001*
	**IA**			0.051	0.414			
	**IR**				0.109			

P values are listed. Bold font indicates group and italics denote p< 0.05 (Student's unpaired *t* tests).

In human subjects, skin thickness increases with age until ∼25 years old and decreases thereafter [5]. By contrast, when hair-cycle stages were held constant, no significant differences in mechanical parameters were observed between young adult and intermediate groups, indicating that age alone does not govern skin mechanics in mice (Figures 4D–F; Table 2). Thus, we conclude that hair-cycle stage dominates skin mechanical properties in adult mice less than 20 weeks of age.

### Skin location impacts thickness, stiffness and elasticity

We next asked how skin mechanical properties varied across anatomical sites by comparing three skin areas on the hind limb. Qualitative observations indicated that proximal skin was thicker than distal areas and quantitative histomorphometry suggests that this was due to differences in dermal thickness (Figure 2).

**Figure 5:**
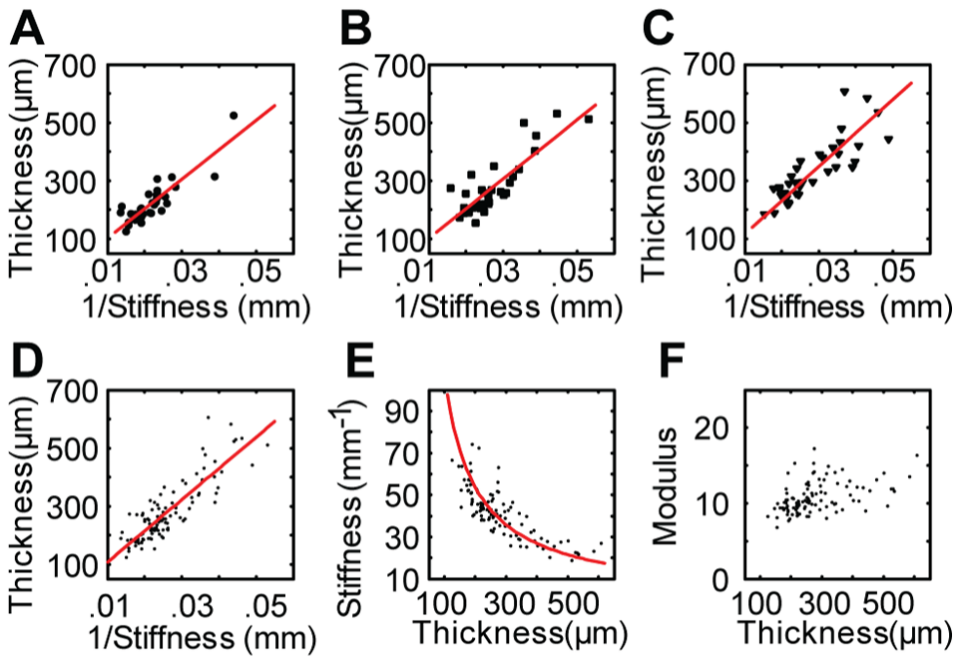
Hyperelastic material properties at three hind-limb sites. Plots show thickness versus the reciprocal of skin stiffness (1/p) for distal (**A**) off nerve trunk (**B**) and on nerve trunk (**C**) groups. Solid lines are linear regression curves, R^2^ = 0.760, 0.722 and 0.686 for a, b and c respectively. The slope of the regression gives the elastic modulus coefficient q (10.14 for Distal, 10.17 for OffNT and 11.58 for NT). (**D)** Linear regression (line) for all skin samples between 1/p and thickness returns thickness  = 10.745/p, R^2^ = 0.74. (**E)** Observed reciprocal relationship between stiffness and skin thickness (*l*_0_) across the pooled dataset of skin specimens. (**F**) Plot of elastic modulus (q) versus thickness for all specimens, demonstrating q is independent of thickness.

Our quantitative mechanical measurements confirmed that distal hind-limb skin was thinnest, proximal skin adjacent to the saphenous nerve (offNT) was intermediate and proximal skin over the saphenous nerve (NT) was thickest (Figure 4G and Table 2). Moreover, we found that anatomical location significantly impacted skin stiffness, with distal skin greater than either proximal location (Figure 4H; p-value<10^−3^, Table 2). The elastic modulus was greatest for skin sites on the saphenous nerve trunk, suggesting that blood vessels and nervous tissue are less compliant than skin (Figure 4I; p<10^−3^, Table 2). For each site, we also plotted the population's elastic modulus as the slope of the regression line between thickness and the reciprocal of stiffness (Figure 5A–C). These data also indicate that the modulus is greatest on the nerve trunk; therefore, changes in thickness and stiffness across locations did not counteract each other as they did across physiological groups to maintain a consistent modulus (Figure 4I). Collectively, these findings reveal that the mechanical properties of adjacent skin areas can differ to a suprising degree. Furthermore, the stiffness of fascial structures, such as blood vessels and peripheral nerves, can significantly impact the mechanics of full-thickness skin.

### Skin mechanical properties differ between individuals

Finally, we asked how skin mechanical properties vary between individuals at a given physiological state. As skin mechanics did not differ significantly between young adult and intermediate age groups, samples were combined into active and resting hair-cycle phases for the purpose of analyzing mouse-to-mouse differences. We observed less variation in skin thickness within the active (314±76 μm, CV = 0.242) and resting groups (235±56 μm, CV = 0.238) than within the mature group (333±145 μm, CV = 0.435). On average, the skin's elastic modulus was similar across all groups (active: 11.52±1.45, CV = 0.126; resting: 10.4±2.1, CV = 0.206; mature: 11.0±2.1 mm^−1^, CV = 0.188).

The high degree of within-group variability in thickness and stiffness suggests intersubject differences that were not systematically examined in this study. Repeated measurements on individual specimens demonstrated that measurement and analysis techniques were highly reproducible (CV = 0.005 for thickness, 0.007 for stiffness and 0.002 for modulus; n = 3 replicates). Thus, we conclude that the observed differences reflect real biological variability. We attempted to reduce variability by focusing on female littermates bred and reared with identical diets and housing conditions. Factors that might have nonetheless contributed to between-animal differences include the use of outbred BDF1 mice, small differences in the locations of the specimens sampled (which could not be controlled on a sub-millimeter scale) and age differences, which were only tracked to the level of a day.

A key finding of this systematic survey is that skin thickness and stiffness vary inversely (Figure 5D–E), resulting in a consistent elastic modulus thoughout the hair cycle and with body-weight changes (Figure 5F). These skin material properties were quantified for the first time 1) under compression, 2) for freshly excised tissue and 3) where body weight, hair cycle phases, maturity level and skin site were methodically varied. In adult mice less than 20 weeks of age, fluctuations in skin stiffness and thickness were largely due to synchronized hair cycles. In ‘middle-aged’ mice, these fluctuations instead correlated with body weight, which most likely reflects dermal fat thickness. In addition to these dynamic changes that depend on physiological state, the mechanics of hairy skin differs markedly with anatomical location, including position relative to fascial structures such as nerves and blood vessels.

Over the entire population, we observed that the skin stiffness coefficient q is a reciprocal function of skin thickness (*l_0_*; Figure 5E), whereas elastic modulus is relatively constant across age, hair cycle and body weight (q = 10.745, R^2^ = 0.74; Figure 5D, F). This finding indicates that, although skin structure changes dramatically throughout adult life, mouse skin at a given location maintains a constant elastic modulus to compression due to counteracting changes in thickness and stiffness. By contrast, previous studies using tensile tests, which measure the stretch of collagen bundles, reported a direct correlation between age and elastic moduli [6]. The compensation that occurs to maintain a constant skin modulus might allow an animal to maintain consistent tactile sensitivity throughout the course of its life.

Our work complements previous studies of how age-related skin mechanical properties change under conditions of tensile loading [6], torsional loading [5] and by using a cutometer [7]. In agreement with our findings, *in vivo* measurements from human subjects aged 18–65 found that skin mechanics differed between body sites [7]. Notably, Krueger and colleagues found that some skin elasticity parameters were highly correlated with age, whereas others were not. Our findings extend these studies by directly measuring skin thickness and identifying a correlation of skin biomechanics with respect to body weight.

Previous studies of the skin under compression are limited to micro-scale indentations [27], [28] and a single-specimen study of pig dorsal skin [2]. Our results using mouse populations agree with these studies regarding the skin's highly non-linear characteristics. Moreover, our results confirm values measured from pig skin (initial Young's modulus: 3.81 kPa for mouse and 7.34 kPa for pig; Young's modulus at 25% strain: 31.78 kPa for mouse and 37.97 kPa for pig skin). These findings demonstrate that the skin's compressive modulus is similar across mammalian species.

We found that the biomechanics of skin under compression differs markedly from the skin under tension. Although the Young's modulus at 5–10% strain in tension (5 kPa [13]) is similar to that measured here in compression, the modulus at 20–30% strain in tension increases by approximately two orders of magnitude (∼6 MPa [29]).

The observed differences in the skin's response to compression versus tension are quite significant for studies of tactile sensation, since many mechanoreceptors respond selectively to deformation caused by compression at the skin's surface [16]–[21]. Thus, previous tension-based studies do not provide an accurate description of skin mechanics for the skin's sensory functions. One gentle touch receptor that responds only to compression is the slowly adapting type I afferent. Preliminary work of the authors modeling the skin's role in mechanosensation used the mechanical measurements in this study to demonstrate that a stable modulus helps maintain consistent SAI afferent responses when skin thickness changes [30]. That work suggests that *in vivo*, mammals might control stimulus intensity using force instead of displacement, which would be an important consideration in neurophysiological experiments and haptic interface design in order to deliver consistent stimuli to diverse end-users.

### Conclusions

Our systematic analysis of compressive tissue biomechanics provides insight into the structural features and physiological states that govern skin behavior under mechanical stress. This study focused on hyperelasticity, which consititutes the time-independent component of skin mechanical properties. Future analysis is needed to define time-dependent viscoelasticity [3]. These findings set the stage for future investigations of skin aging, UV damage and sensory signaling, and will inform development of new skin-targeting therapies such as surgicial procedures and drug delivery systems.

## Materials and Methods

### Compression Test Apparatus

A custom-built apparatus was used to perform uniaxial compression tests of cylindrically cut skin samples. Equipment consisted of a vertically oriented load sled with a compression tip whose position was tracked by a laser and force by a load cell (Figure 1B). The compression tip was an aluminum plate, 3 mm thick and 2.54 cm diameter, connected by a rod to a load cell (Honeywell, Miniature Model 31, Columbus, OH) with full capacity of 2.45 N. The load cell was mounted to the motion-controlled sled (motion controller: Newport, Model ESP300, Mountain View, CA; linear stage: Newport, Model ILS100). The tip compressed the skin specimens against a rigid plate parallel to the tip's surface. A laser displacement sensor (optoNCDT Model ILD 1402, Micro-Epsilon, Raleigh, NC) was used to measure displacement with resolution of 1 μm. Data were logged at a 1-kHz sampling frequency. A closed-loop system was integrated to control the temperature of the rigid plate using BASIC Stamp microcontroller module (Parallax Inc., Rocklin, CA).

### Animals

Animal use was conducted according to the National Institutes of Health *Guide for the Care and Use of Laboratory Animals* and was approved by the Institutional Animal Care and Use Committee of Columbia University (protocol AC-AAAC1561). Euthanasia was performed under isoflurane anesthesia and every effort was made to minimize suffering. A total of 24 adult female mice (BDF1 background) were sacrificed at ages ranging from 5.7–34.3 weeks (Figure 3). Hair-cycle stages and skin maturity levels were determined based on age and histological criteria (skin pigmentation and hair-follicle morphology). Animals at 5.7–6.9 weeks were identified as group Young Active (YA) and at 9.0–10.3 weeks were identified as in group Young Resting (YR [25]). Similarly, animals at 12.6–12.7 weeks were identified as Intermediate Active (IA), based on published ages and duration of anagen, and confirmed by skin pigmentation; 16.9–19.3 weeks were identified as Intermediate Resting (IR), by examining their skin as they are known to be in a phase of mosaic hair cycling. Mature mice were designated as 26.3–34.3 week animals (M).

### Dissection

Hair was removed with a commercial depilatory cream (SurgiCream, Ardell International, Commerce, CA) and then specimens of hairy skin were dissected from the mouse hind limb using protocols described for skin-nerve preparation recordings [16]. Specimens were constantly hydrated with physiological synthetic interstitial fluid (SIF) throughout experimentation. Freshly isolated skin specimens were used for mechanical measurements within ∼1.5 h of dissection. Skin punches were obtained using 6 mm diameter biopsy punch (Acuderm Inc., Ft. Lauderdale, FL; Figure 1C). Sampling sites were selected because they contain tactile end organs and appear to be categorically differentiable in terms of thickness and stiffness.

### Histology and quantitative morphometry

Skin specimens were harvested from euthanized animals and depilated as described above. After dissection, skin was fixed overnight in 4% paraformaldehyde and stored in 70% ethanol for 24–72 hours. Tissue was embedded in paraffin and sectioned at 5 µm for Hematoxylin and Eosin (H&E) staining. Paraffin embedding and H&E staining was performed by the Skin Disease Research Center (SDRC) Tissue Culture and Histology Core at Columbia University. Samples were imaged with brightfield microscopy (Axioplan2, Zeiss, Thornwood, NY; 10X, 0.45 NA lens) in the SDRC Advanced Imaging Core. Six epidermal and six dermal thickness values were measured from three histological sections per hind-limb site (Distal, OffNT, NT; n = 3 mice per group; ages 6, 12 and 15 weeks). Means of the six measurements from each section was used for statistical analysis. Measurements are reported in pixel values.

### Skin Test Procedure

Freshly excised specimens from five hair cycle phases, three maturity levels and three skin sites were studied. These included 35 distal, 34 OffNT and 36 NT specimens. Maximum indentation depths were determined by manually searching for an instantaneous reaction force of approximately 2 N, which generates a strain level of ∼25%, matching indentations in electrophysiological recordings [16]. The starting position of the compression tip was above the skin surface. Each skin specimen was placed flat under the center of the tip. Specimens were displaced with a constant ramp-up speed of 10 μm·s^−1^ while the reaction force was logged. SIF was added via an eye dropper to maintain skin hydration.

### Calculation of Material Properties

**Figure 6:**
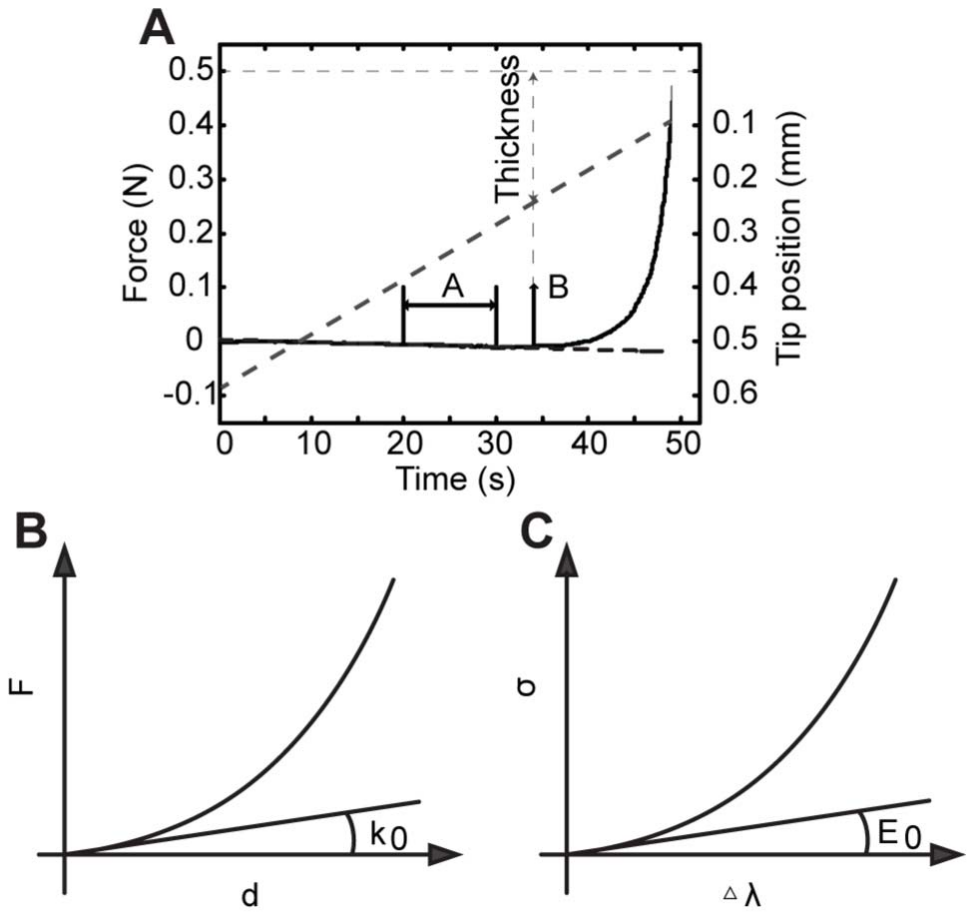
Summary of procedures for measuring and calculating hyperelastic skin material properties. (**A**) Example plot of force and displacement versus time. Illustration of SIF compensation of force traces, where interval A is the interval picked for curve-fitting in compensating error caused by SIF, and contact point B. Solid black line: force. Dashed gray line: displacement. Dashed black line: curve fit for SIF compensation. (**B**) Schematic force vs. displacement plot. k_0_ is the slope of the linear region, which indicates the initial stiffness of skin. (**C**) Schematic stress vs. change in stretch plot. E_0_ is the slope of the linear region, which indicates the initial Young's modulus of skin.

Force versus displacement data were first corrected for noise reduction and then converted into stress versus stretch-change plots. Each force trace was fitted using a cubic spline function for noise reduction and smoothing. Next, the whole curve was compensated for the linear offset caused by reaction force from SIF. This was performed by manually choosing a time range before contact (*i.e.,* 5–10 s window before the force rose markedly; interval A in Figure 6A) and then the whole curve was offset by the line fitted to this interval of data. Before skin compression experiments, plate-contact position was determined by moving the compression tip toward the temperature-controlled plate until a change in force was detected. The vertical position of the tip was recorded by the displacement sensor. After skin was placed on the plate, any force magnitude larger than a threshold (F_T_) of 0.01 N, which is clearly above measurement noise levels, was recorded as skin-contact position. The difference between plate-contact position and skin-contact position denotes skin thickness (*l_0_*). This method was inspired by Wu *et al.* [2] and is more accurate than measurements by caliper [11] or dial micrometer [31] because it removes the observer-dependent variability inherent in these methods.

We next converted the raw data (force versus displacement) to stress and stretch. Stretch (λ) was calculated by deformed thickness (*l*) over original thickness (*l_0_*) [32]:
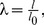
(1)


For convenience in calculation, the change in stretch during compression is defined as:
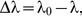
(2)in this case λ_0_ = 1. Similarly, compressive stress was defined as positive and calculated using force over area, which was observed to be approximately constant by a camera placed beneath the sample during test runs.

Third, we sought appropriate form of functions and parameters to characterize the constitutive equations for skin material. After testing different candidates, we found the modified exponential function [33] gave the best fits. A single parameter curve fitting was used to fit both force versus displacement curves and stress versus stretch-change curves. Force versus displacement traces (Figure 6B) were approximated using Equation (3),
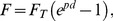
(3)where F is the reaction force at the compression tip, F_T_ denotes the contact force threshold, the exponential linear coefficient p indicates the non-linear stiffness of the skin (referred to as stiffness exponent, or stiffness) and *d = l_0_*-*l* represents displacement into skin, which was calculated from the position of the compression tip at the time when the force transducer reading rises above the pre-set contact threshold F_T_. Per Eqn. (3), the controlled and measured variables for each skin compression test are d and F, whereas F_T_ is a constant. Once d and F were collected over the entire displacement sequence for a skin specimen, we attained a single stiffness exponent p to characterize the mechanical behavior of that skin specimen. We next compared stiffness exponents across the data pooled from different skin specimens.

Similarly, Equation (4), was used to approximate the stress versus change in stretch curve (Figure 6C),
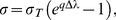
(4)where 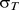 is the stress value at contact threshold and is obtained by F_T_/A, A denotes surface area of the specimen, A  =  πr^2^, and r is the radius of the sample (r = 3 mm), the exponential linear coefficient q indicates the hyperelastic modulus of the specimen (referred to as modulus exponent), σ represents Cauchy stress obtained by F/A and Δλ represents stretch change, with the reference length of the skin thickness. Similarly, modulus is constant with respect to stress and change in stretch; therefore one modulus value is derived per skin specimen. We found that this modulus value differs between specimens.

Two important derivations from the formula above were used for analysis. Detailed derivations are included below. The initial stiffness and initial modulus of skin:
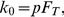
(5)

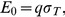
(6)where k_0_ and E_0_ denote the initial stiffness and initial Young's modulus of the skin. These two parameters sufficiently described the material elasticity under small deformations; however, since the skin is highly compliant and hyperelastic, these two parameters are not the best parameters to characterize the skin under compression greater than approximately 5% (Figure 6B–C). Eqn. (5–6) can be acquired by calculating partial derivative of Eqn. (3) and Eqn. (4) with regard to d or Δλ at value 0.

The relationship between the stiffness exponent (p) and the modulus exponent (q):
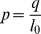
(7)


Recall that *l_0_* is the thickness of the skin. Eqn. (7) can be derived by solving Eqn. (1–4) together.

Curve fitting of force versus displacement and stress versus stretch change was performed via MATLAB (Mathworks, 2011b, Natick, MA). The average resultant R^2^ values for the all fitting was 0.98.

### Statistics

Statistically significant differences between groups were assessed by unpaired Student's *t* tests to examine the effect of hair cycle and skin site on all three mechanical properties (thickness, stiffness and modulus). Unpaired Student's *t* tests were chosen because the data were pooled from unmatched specimens. To study the connection between skin properties and body weight, Pearson tests of correlation were also performed between weight and all three mechanical properties. Statistical analyses of mechanical measurements (Tables 1–2) were performed via MATLAB (Mathworks, 2011b, Natick, MA). Student's two-tailed unpaired *t* test were used to assess quantitative histomorphometry (Prism 5, Graphpad Software, La Jolla, CA).

### Derivation of Equations

#### Derivation of equation 5

Stiffness is defined by force over displacement. Therefore, initial stiffness k_0_ can be derived by:
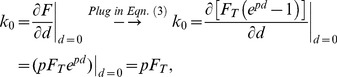
(8)


#### Derivation of equation 6

Modulus is defined by stress over strain. Therefore, initial modulus E_0_ can be derived by:
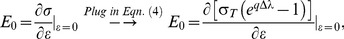
(9)where ε denotes strain. Note that



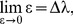
(10)Thus, we have
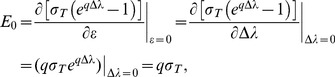
(11)


#### Derivation of equation 7

Divide the RHS and LHS of Eqn. (3) by the RHS and LHS of Eqn. (4) correspondingly, we have
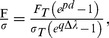
(12)


Note that 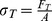, 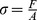. Thus,
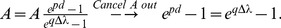
(13)


Organize, we get
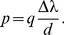
(14)


Plug in Eqn. (1–2), and note that 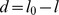,
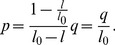
(15)


Which is the same with Eqn (7).
